# Comparison between two different registration protocols for the count of sleep bruxism events in a sample of healthy individuals

**DOI:** 10.1007/s00784-025-06394-2

**Published:** 2025-07-02

**Authors:** Ovidiu Ionut Saracutu, Matteo Pollis, Ludovico Bulferi Bulferetti, Elifnaz Kaya, Edoardo Ferrari Cagidiaco, Marco Ferrari, Anna Colonna, Daniele Manfredini

**Affiliations:** School of Dentistry, Department of Medical Biotechnologies, University of Siena, Siena, 53100 Italy

**Keywords:** Sleep bruxism, Electromyography, Maximum voluntary contraction, STAB, Instrumentally-based assessment

## Abstract

**Objectives:**

The aim of the present research is to compare, in a group of healthy young individuals, the number of sleep bruxism (SB) events with the use of two different electromyographic (EMG) devices, which adopt two different protocols for scoring the events.

**Materials and methods:**

The study was performed on 10 individuals recruited at the University of Siena, Italy. Participants were asked to perform one night of sleep-time recording wearing simultaneously, in their home environment, two different EMG devices, the Bruxoff (Bruxoff^®^, Turin, Italy) and the dia-BRUXO (dia-BRUXO, Biotech-Novations, Sanremo, Italy). SB events were identified by the Bruxoff software algorithm as the EMG peaks of an amplitude that exceeds 10% of the maximum voluntary contraction (MVC) performed by the subject before the start of the registration, and that are preceded by a 20% increase in the heart rate compared to the baseline. Conversely, the dia-BRUXO software identified the EMG peaks exceeding 10% of the maximum contraction (MC) recorded during the entire registration period. Both devices were set to count the events lasting 1–5 s. After each night of recording, data was downloaded through the dedicated software of each device, which generates a report with the number of sleep bruxism events.

**Results:**

All the recruited participants completed the registrations at the first attempt without reporting any detachment of the electrodes. The Pearson test showed a strong correlation between the number of SB events scored by the two devices (*r* = 0.9613, *p* < 0.001).

**Conclusions:**

Within the limitation of this study, our results suggest the existence of a strong correlation between the two different registration protocols for the registration of the number of SB events.

**Clinical relevance:**

The present study suggest that clinicians can adopt easier and user-friendly protocols for the registration of sleep bruxism events, that do not necessarily require a pre-calibration of the device.

## Introduction

In the last decade, there has been an important increase in the number of research papers that focus on bruxism [1]. In 2013, an international panel of experts tried for the first time to provide researchers and clinicians with a universally accepted definition [2]. Five years later, the authors presented two separate and more specific definitions of bruxism, based on the circadian manifestation. Awake bruxism (AB) is defined as a *masticatory muscle activity during wakefulness that is characterised by repetitive or sustained tooth contact and/or by bracing or thrusting of the mandible and is not a movement disorder in otherwise healthy individuals*,* while sleep bruxism (SB) as a masticatory muscle activity during sleep that is characterised as rhythmic (phasic) or non-rhythmic (tonic) and is not a movement disorder or a sleep disorder in otherwise healthy individuals* [3]. Thus, in the current state of evidence, bruxism is an umbrella term for a series of masticatory muscle activities and not a parafunction or a pathological condition [4].

Data on the epidemiology of SB remain ambiguous, with a reported prevalence ranging from 8 to 31.4% for adults and from 3.5 to 40.6% for children [5, 6]. Such high heterogeneity among studies could be explained by the different methodologies adopted by researchers, which were a consequence of the lack of standardization protocols for the assessment of bruxism.

Only recently, the STAB (Standardized Tool for the Assessment of Bruxism) was developed as the first non-stackable multidimensional tool for the evaluation of bruxism [7, 8]. The STAB includes domains to gather data on the assessment of bruxism (Axis A of STAB) and the related risk factors and associated disorders (Axis B of STAB) [9]. Axis A contains three domains to evaluate the presence of bruxism, which can be performed through a subjective-based assessment (SBA), a clinically-based assessment (CBA), and an instrumentally-based assessment (IBA). Remarkably, most of the literature on bruxism is based on the SBA, or on the combination of the SBA and CBA. Nevertheless, experts in the field consider the IBA to be the approach that can provide clinicians and researchers with the most accurate data, reducing recall bias [9].

The STAB proposes two types of IBA, viz., the Ecological Momentary Assessment (EMA) and surface Electromyography (EMG). The EMA has proven to be a tool that provides more data compared to the single self-report SBA [10–13] and is well-accepted by the users [14]. Unfortunately, such technology is based on self-report and can only be used for the assessment of AB, leaving EMG as the only currently available option to measure the full circadian spectrum of bruxism activities, including also SB [15].

The choice of the ideal EMG criteria for SB has been a matter of ongoing debates [16–18], considering the high variability in terms of intensity and duration of the SB events occurring during sleep [19]. The STAB suggests for clinicians two types of solutions [9]:

i) The count of the number of events over 10% of the maximum voluntary contraction (MVC) [20, 21];
ii) The bruxism work index (BWI) and the bruxism time index (BTI). A possible option to assess the BWI is the strategy that was recently suggested by Colonna et al., who calculated the percentage of muscle work during bruxism-related masticatory muscle activity (MMA) compared to the potential work that could be exerted if the highest peak of power registered during the 24 h had been kept unvaried during all the bruxism episodes. Similarly, the BTI can be viewed as the percentage of time with bruxism-related MMA with respect to the total recording time, respectively [22, 23].

Of the two above strategies, the most diffused method is the adoption of a threshold at 10% of the MVC to identify the number of bruxism events during sleep [24, 25]. Such strategy was based on some pioneer data by Lavigne et al., who assessed the correlation with a battery of clinical and anamnestic findings in a group of volunteers [26]. Nevertheless, the STAB does not specify how to determine the MVC. Currently, clinicians can adopt devices that propose different methods to assess the MVC. Some manufacturers suggest clinicians to ask the patient, prior to the registration, to perform three MVCs separated by 10 s of rest. The device then uses the highest MVC value of the three attempts as a reference value and identifies as bruxism events all the muscle contractions exceeding 10% of that MVC [27–29]. Some other devices do not require to perform a dedicated process before the start of the registration and automatically take as a reference value the highest maximum contraction (MC) registered during the whole monitoring period [14, 22, 23]. Questions and doubts may arise among practitioners about which of the two protocols should be used; as of now, there is no evidence of a comparison between the two approaches.

To shed more light on the topic, the aim of the present research was to compare the number of bruxism events occurring during sleep, with the use of two different devices, which adopted the two different protocols that are currently available, viz., the highest maximum contraction (MC) and the induced MVC, as sources of the 10% threshold values to identify SB events.

## Materials and methods

### Participants recruitment

The study was performed on 10 individuals recruited at the University of Siena among patients attending the dental department for dental check-ups. Participants were required to be in good general health and to be available to perform one night of SB monitoring with EMG. Exclusion criteria were any ongoing medical, pharmacological, or dental treatments, sleep disorders (e.g., insomnia, periodic leg movements), or neurological/psychiatric conditions. All the subjects were not under medications at the time of recording and were not under the effects of alcohol or caffeine.

**Fig. 1:**
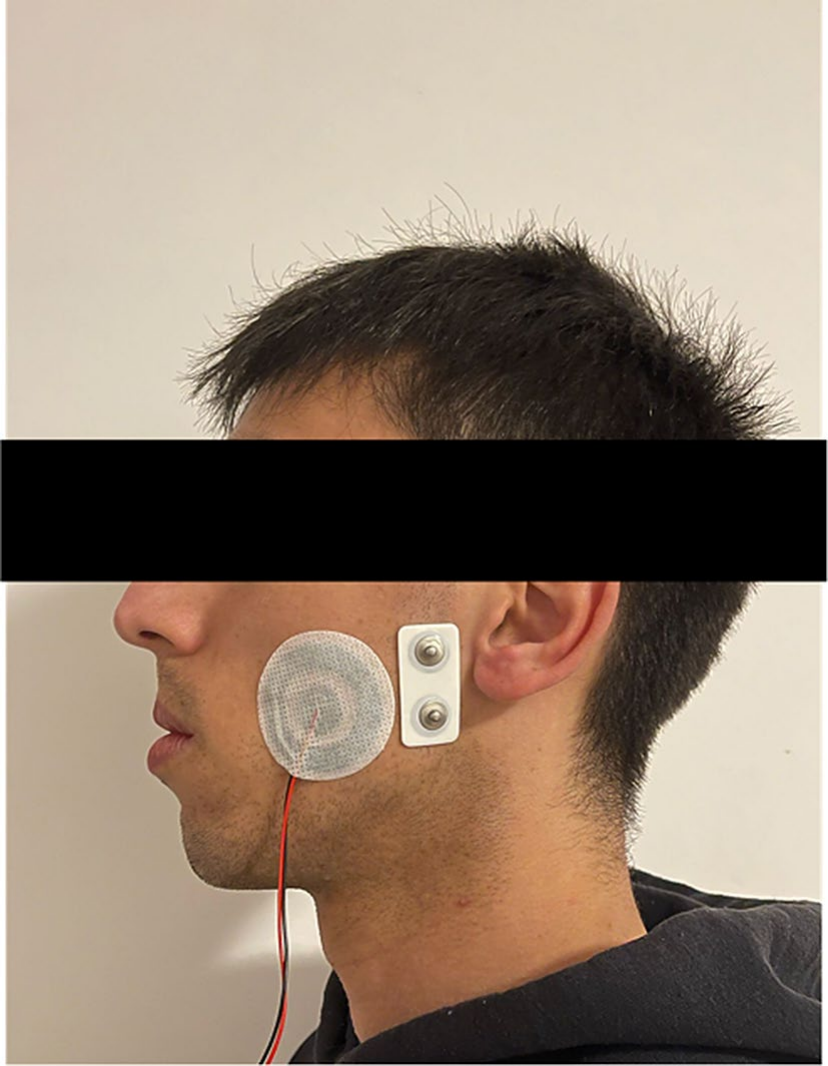
Photo of a patient wearing the electrodes of the Bruxoff (AgCl concentric electrodes (Code^®^) with a 16 mm radius) and the dia-BRUXO (bipolar AgAgCl electrodes with solid gel, with interelectrode distance of 22 mm) devices, simultaneously

**Fig. 2:**
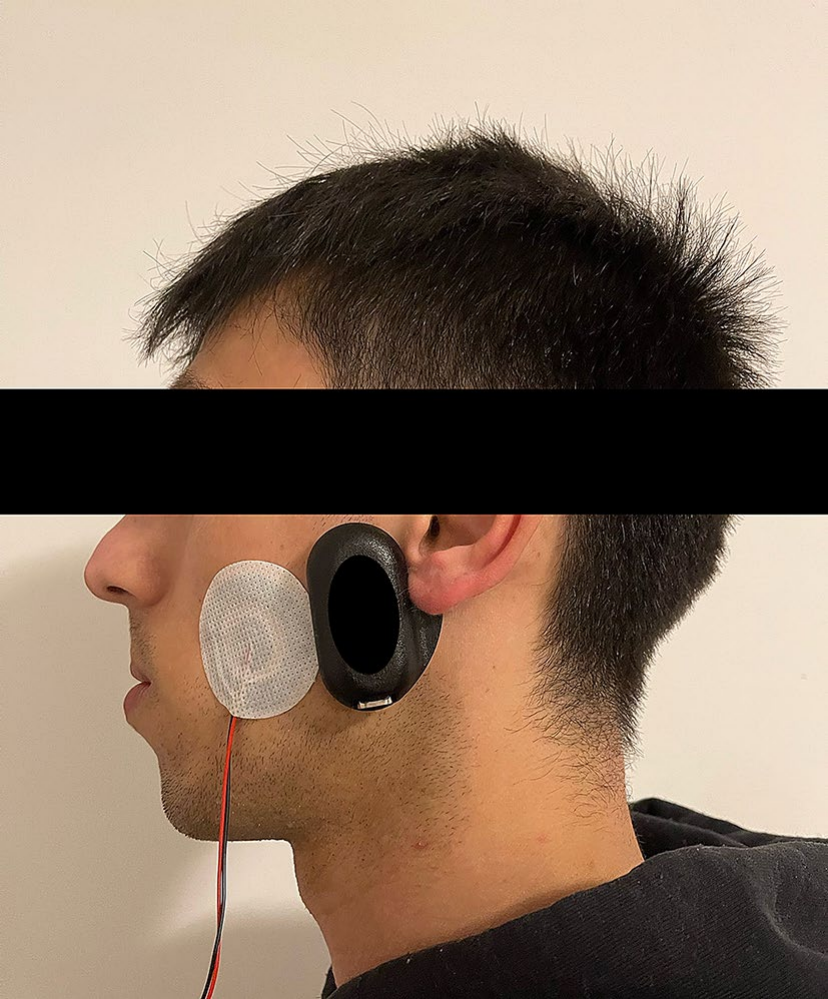
Photo of a patient wearing both the Bruxoff (Bruxoff^®^, Turin, Italy) and the dia-BRUXO (dia-BRUXO, Biotech-Novations, Sanremo, Italy) devices simultaneously

Participants were required to perform one night of sleep-time recording wearing both the Bruxoff (Bruxoff^®^, Turin, Italy) and the dia-BRUXO (dia-BRUXO, Biotech-Novations, Sanremo, Italy) devices simultaneously in their home environment (Figs. 1 and 2). For the placement of the electrodes, the instructions provided by the manual of the devices were followed. The supervision was done by the same trained operator who placed the electrodes on all participants (L.B.B). After each night of recording, data was downloaded through the dedicated software of each device, which generates a report with the number of SB events. Considering that the aim of the present study was to compare two different protocols for the registration of sleep bruxism events and not their absolute validity in assessing bruxism, it was not considered necessary to perform an accommodation night.

All individuals gave their informed consent in accordance with the Helsinki Declaration and understood that they were free to withdraw from the study at any time. The research protocol was approved by the Institutional Review Board of the Orofacial Pain Unit, University of Siena, Siena, Italy (#0125–2024).

### Bruxoff registrations

Bruxoff (Bruxoff^®^, Turin, Italy) is a portable electromyographic device with three channels that can record the masticatory muscle activity of both masseters as well as the heart frequency. The three signals were sampled at 800 Hertz (Hz),with 8-bit resolution. Data were stored on a MicroSD card as a binary file. The EMG and the electrocardiography (ECG) channels were filtered between 10 and 400 Hz with a gain of 4300 and between 15 and 160 Hz with a gain of 700, respectively. The muscle activity was detected with disposable bipolar AgCl concentric electrodes (Code^®^) with a 16 millimeters (mm) radius [30]. Such electrodes, besides being easy to apply over the area of interest, were demonstrated to avoid problems concerning the orientation of the electrode and limit EMG crosstalk [31]. Electrocardiographic activity is instead measured through a disposable bipolar electrode placed on the left side of the thorax, 5–10 cm below the sternum.

For the application of the registration protocol, the authors followed the instructions provided by the device manufacturer. The subject is required to perform five tapping movements before sleep and at awakening. Such tapping movements allow the device to recognize the beginning and the end of the registration. To calibrate the device to the individual masticatory muscle strength, subjects are asked to perform three MVC attempts. The episodes are required to last at least three seconds and be separated by a pause of 10 s. The highest value that was reached is taken as a reference to normalize the SB event. The device records a bruxism event, an episode over 10% of the MVC lasting 1–5 s, preceded 1 s before by a 20% increase in the heart rate compared to the baseline [32].

At the end of the registration, the main investigator (L.B.B.) detached the device from the participant and connected it to the computer using the device’s dedicated software (Bruxmeter software^®^). The software can automatically score the number of bruxism events for each masseter muscle, according to the afore-mentioned criteria.

### dia-BRUXO Registrations

The dia-BRUXO device (dia-BRUXO, Biotech-Novations, Sanremo, Italy) is a portable electromyographic device with one channel that can record the masticatory muscle activity of the left masseter. According to the manufacturer’s indications, the muscle activity is detected by a disposable electrode with solid gel and AgAgCl sensors. Between the two electrodes, there is a space of 22 mm to reduce crosstalk [33]. The signal is elaborated through a three-stage analog circuit, an amplification circuit, an active bandpass filter (between 110 Hz and 550 Hz), and an RMS (root-mean-square) integrator. The analog information is digitalized in the processor by a 12-bit analog/digital converter (4096 discriminating levels), with an acquisition every 100 mS.

To activate the registration of the device, it must be first connected to the dedicated software (dia-BRUXO software^®^) installed on a computer with a cable. Before starting the registration, the software requires the investigator to insert the subject’s personal data (date of birth, gender). Moreover, the software of the device allows the user to choose the percentage of the spontaneous MC that is used to identify a bruxism event (i.e., 5%, 10%, or 20% of the MC). For this investigation, 10% of the MC, lasting 1–5 s, was set as a threshold. After setting all the parameters, the main investigator (L.B.B.) detached the device from the computer and placed it over the skin overlying the left masseter muscle after the Bruxoff device and electrodes were already set and applied over the surface of the same muscle. In such a way, both devices were able to detect the number of contractions of the left masseter muscle, using the same threshold value.

After the completion of the registration, the device was detached and connected to its software, which generates a report with the number of sleep bruxism events occurring during sleep.

### Statistical analysis

After data collection, descriptive analysis was performed for all the variables. Pearson‘s correlation test and the Bland-Altman plot were used to quantify the magnitude and direction of concordance and the agreement between the two devices in measuring the number of sleep bruxism events occurring during sleep. The level of statistical significance was set at *p* < 0.05. The data contained in the two-report generated by the devices for each subject were inserted into an Excel document. Statistical analysis was performed with Microsoft Office Excel 2021 (Los Angeles, CA, USA).

## Results

**Fig. 3:**
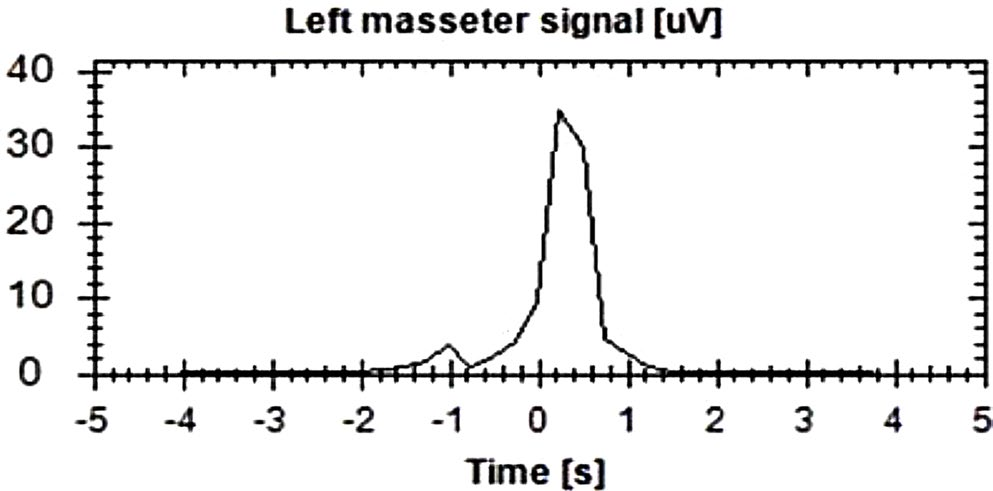
An example of a sleep bruxism event detected by Brux-off. The x-axis indicates the duration of the event in seconds, while the y-axis shows the intensity of the signal of the left masseter muscle in microvolts. The second 0 indicates the instant in which the device started to detect the activity of the left masseter muscle over 10% of the maximum voluntary contraction (MVC). In this case, sleep bruxism event had an intensity between 30 and 40 microvolts

**Fig. 4:**
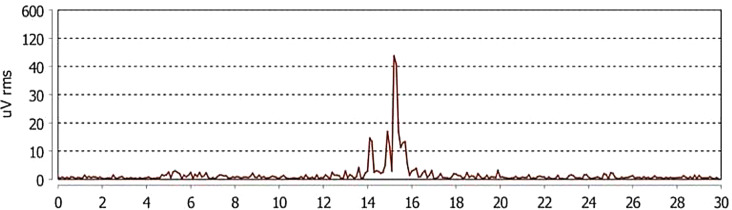
Example of sleep bruxism event detected by dia-BRUXO. The x-axis indicates the duration of the event in seconds, while the y-axis shows the intensity of the signal of the left masseter muscle in microvolts. The sleep bruxism event was detected between the 14th and 16th second of the registration, with an intensity that is higher than 40 microvolts

**Fig. 5:**
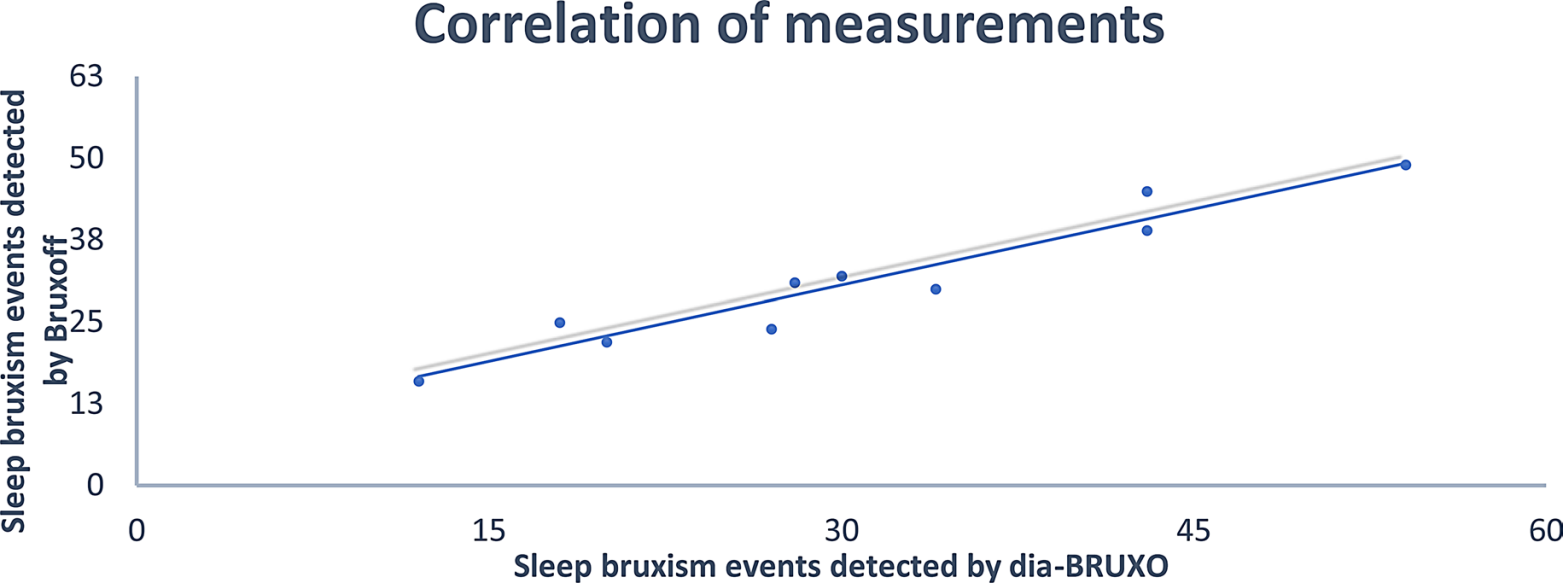
Correlation between Bruxoff and dia-BRUXO detection of sleep bruxism events (*r* = 0.9613, *p* < 0.001)

All the recruited participants, five males (mean age 21.9 ± 1.3, range 21–24 years) and five females (mean age 21.2 ± 1.1, range 20–22 years), completed the registrations at the first attempt without reporting any detachment of the electrodes. Moreover, the participants reported no considerable sleep interruption that might have influenced the outcomes with any of the two devices. The average sleep time was 7.8 h ± 0.7, range 7 to 8 h. The Bruxoff device registered an average number of sleep bruxism events of 31.3 ± 10.41, while the dia-BRUXO scored 30.9 ± 12.92 (Figs. 3 and 4). The Pearson test showed a strong correlation between the number of sleep bruxism events recorded by the two devices (*r* = 0.9613, *p* < 0.001) (Fig. 5).

**Fig. 6:**
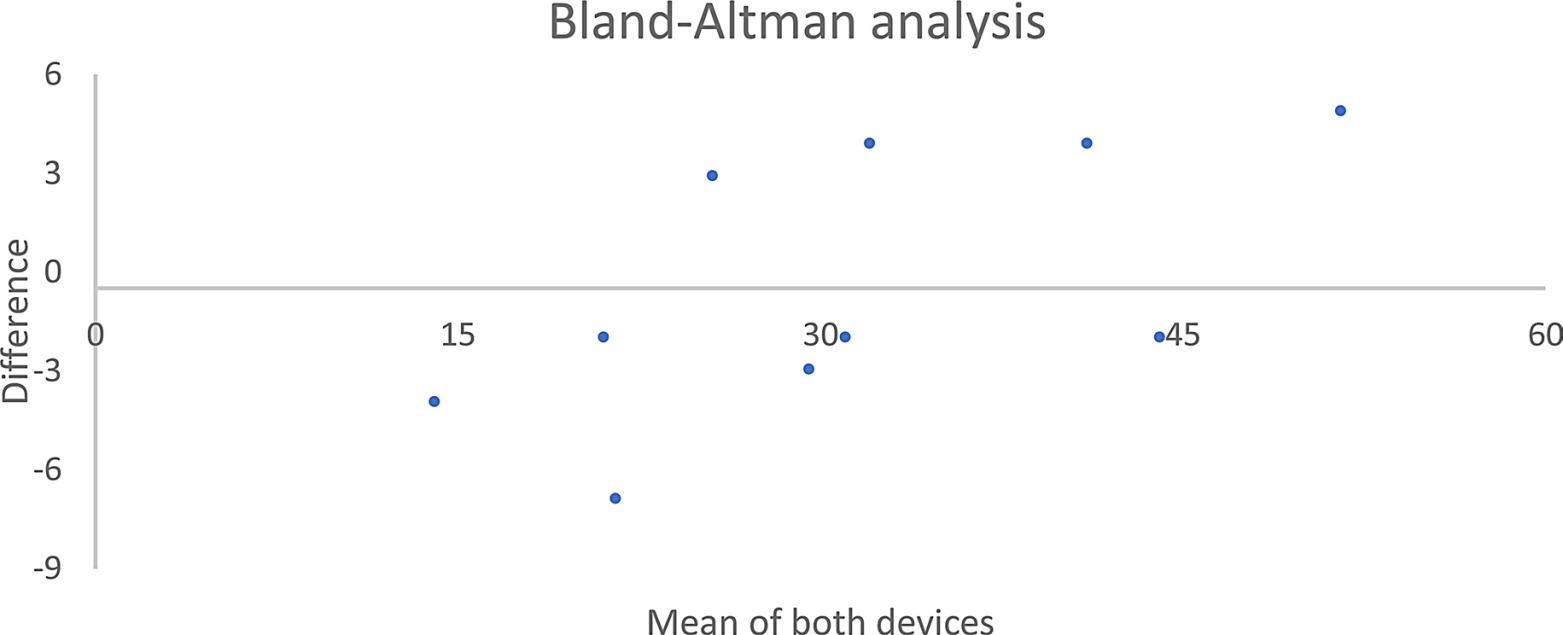
The Bland-Altman plot showed a high level of agreement between dia-BRUXO and Bruxoff, with a bias of -0.4 between the two measurements and a standard deviation of bias of 4.1. The 95% confidence interval ranged between − 8.4 and 7.6 on the vertical axis. The horizontal axis of the graph corresponds to 0 (i.e., no difference between the two protocols of registration) and 95% limits of agreement ranging between − 8.4 and 7.6

The Bland-Altman plot showed a high level of agreement between the two devices, with a bias level of -0.4 between the two registration protocols (Fig. 6).

## Discussion

In the last few years, the diffusion of surface EMG among researchers and clinicians involved in the bruxism field has increased [34]. While its utility for chairside evaluation in the field of temporomandibular disorders (TMDs) and orofacial pain has always been questioned [35, 36] due to the inability to add relevant information compared to the clinical examination [37], EMG remains indeed the main type of instrumentally-based assessment strategy for bruxism [9, 38]. The most widely used assessment method to assess SB is the count of events identified as a percentage of a specific MVC. Currently, on the market, there are devices that adopt 10% of the induced MVC [26, 28] and others that derive thresholds based on the spontaneous MC that is recorded during the monitoring period [22, 23]. The aim of the present investigation was to compare two different EMG registration protocols for the assessment of SB with the use of two EMG devices currently available.

In a sample of healthy young individuals, the present study found a strong significant correlation (*r* = 0.96) between the two different protocols, indicating that adopting one or the other approach does not influence the assessment of bruxism in terms of event counts. Such finding would suggest that, before starting an EMG recording for SB assessment purposes, the patient is not necessarily required to perform the three sets of artificial MVC attempts before the procedure, considering that using the maximum spontaneous MC exerted during the recording period gave very similar results. Additionally, it is also worth mentioning that compared to the dia-BRUXO, the Bruxoff incorporates an electrocardiographic assessment and adopts a software algorithm that recognizes as SB events all the EMG peaks exceeding 10% of the MVC that are also accompanied by a 20% increase in the heart rate compared to the baseline. Despite this additional feature, the number of SB events detected by the two devices correlates strongly (*r* = 0.9613), suggesting that the electrocardiographic analysis does change the number of events detected by the EMG. Given the high rate of agreement between the two approaches, it could be hypothesized that the use of the spontaneous MC, being more user-friendly, would facilitate the in-home recording of the masticatory muscle activity, not requiring the calibration of the induced MVC.

Experts in the fields agree that the instrumental assessment of bruxism is the type of evaluation that provides bias-free information on the frequency of bruxism and should represent the future [9]. However, there is a lack of scientific papers on IBA in the literature. Moreover, the few papers available are characterized by small sample sizes [2, 3, 9, 24]. The main reason for such inevitable shortcomings is related to the high economic cost and the difficulty to perform such registrations, which require home assistance of a healthcare provider to make sure that the registrations are properly conducted. In the absence of a healthcare provider, patients often struggle to self-perform a registration. Clinically, it is not uncommon for practitioners to rent an EMG device to a patient who spends nights with the device without any certainty about the proper positioning of electrodes or control of other technical factors. Such issues may also reduce the willingness and the patient’s compliance to re-perform them. Thus, techniques and suggestions that simplify the registration protocols are very much needed to gather more data on bruxism in the research setting and to assist clinicians in the IBA of bruxism. Considering that this is the first investigation to perform this type of analysis between two different registration protocols, a comparison with other studies was not possible. Importantly, this paper did not analyze the accuracy of the protocols that are currently available for assessing SB. Indeed, it is worth mentioning that none of the devices have been tested to determine their reliability in assessing sleep bruxism properly, which is still a matter of debate [17].

Within the current knowledge, the identification of SB events as a specific percentage of an MVC remains the most used approach [25]. According to experts in the field, such an approach inevitably has certain limitations due to the impossibility to evaluate the full spectrum of bruxism behaviors with software algorithms just based on the count of events [9]. Evidence shows that the MVC has a high intraindividual repeatability [20, 39], making it a potentially useful reference to discriminate SB events. Nevertheless, while some instrumental studies proved the validity of such criteria, showing that self-reported bruxers have a higher number of SB events compared to controls [25, 32, 40, 41], some other investigations unveiled their limits [42–45], raised two main limitations of these criteria.

The first limit is related to the lack of correlation between a specific number of SB events per hour or per night and clinical findings, such as TMDs-related pain [46–48] and tooth wear [49, 50], which are the main clinical consequences of bruxism and the main reasons for patients to seek treatment. Most of the research in the field of the electromyographic assessment of SB has been performed with the hypothesis that a specific number of bruxism events should be used as a threshold to discriminate between healthy individuals and patients. However, such a hypothesis implies that in all individuals, the same type and pattern of masticatory muscle activity and the amount of muscle work inevitably cause similar clinical consequences, ignoring the biological variability and host response [51]. On the contrary, what seems to play a more important clinical role is the background masticatory muscle activity, which, despite having lower intensity than a specific threshold of the MVC and not counting as a bruxism event, can instead cause a significant overload of the stomatognathic system structures [52].

The second limit of the protocols based on the SB counts of events is related to their inability to discriminate between the different types of masticatory muscle activities and classify them according to intensity and duration. As well known, bruxism is a multifaceted phenomenon characterised by a multitude of conditions distinguished, amongst the others, by the quality (i.e., clenching, grinding, bracing, tooth contact) and frequency (tonic, phasic, mixed) of the masticatory muscle activity (3). The mere use of the aforementioned criterion does not allow to discern the variability of such a multifaceted condition and phenotype bruxism, also carrying the risk of not matching with the current definition. As an example, long-lasting, prolonged, low activity muscle contractions, which may be clinically relevant, are not necessarily detectable just by counting the number of events over a certain threshold occurring during a specific timespan [53, 54].

For these reasons, the next step of instrumental assessment of bruxism should rather include the types and/or the total amount of masticatory muscle activity, which are potentially better proxies of the musculoskeletal work [55]. Currently, the literature is not supportive of definitive data on the natural fluctuation of bruxism activities, so gathering more data on this issue is fundamental to understand the clinical relevance of any observed change over time. Such fluctuations can only be measured by indexes that assess the total amount of masticatory muscle activity and bruxism-related activity during the 24 h. For such purposes, in the last years, some indexes were introduced in the literature, such as the BWI and the BTI proposed in the STAB [8], as well as the non-functional masticatory muscle activity work index (nfMMA-WI) and non-functional masticatory muscle activity time index (nfMMA-TI) [23]. The nfMMA-WI measures the percentage of muscle work over twice the baseline EMG levels with respect to the potential work that could be exerted if the highest peak of power registered during the 24 h had been kept unvaried during all the recording periods. Conversely, the nfMMA-TI is defined as the percentage of time that featured EMG levels over twice the baseline with respect to the total recording time. Whether such indexes are a better numerical representation of the full spectrum of bruxism activities has yet to be tested in the clinical setting. Future studies should also test the possible correlation between such indexes and the count of bruxism events as per the strategy adopted so far, possibly testing their accuracy in predicting health risk [56].

Another contribution in the field seems to come from the use of artificial intelligence (AI) and machine learning to detect specific types of bruxism events. Few pilot studies have shown that AI can provide detailed information on the state of the masticatory muscle in real-time and analyze the mandibular movements during still. Although such technology is promising, it is still in its infancy [57, 58]. Within these limitations, clinicians can only use specific threshold values to detect bruxism events also as part of protocols to monitor bruxism over time [26].

The main limitation of the present investigation is the small sample size, which is quite common in studies on the electromyographic assessment of SB. In the present case, the difficulty of recruiting participants was enhanced by the fact that individuals were required to wear two EMG devices at the same time. Moreover, to reduce the risk of bias, these kinds of studies should be performed on healthy individuals who are not under the effect of any medication or treatment, which is a condition that is not easy to find. In the future, the intra-individual repeatability of the two devices and their correlation must be tested to overcome the single night of monitoring featuring this investigation. Future studies should also assess whether there is any significant difference in the comfort and compliance of wearing one or the other device.

## Conclusions

Within the limitation of this study, our results suggest the existence of a strong correlation between two registration protocols for the registration of SB events, that is, the identification of SB event as a muscle activity over 10% of the induced MVC or as a muscle activity over 10% of the spontaneous MC. Therefore, the use of the spontaneous MC, being more user-friendly, would facilitate the in-home recording of the masticatory muscle activity, not requiring the calibration of the induced MVC.

## Data Availability

No datasets were generated or analysed during the current study.
